# A framework towards digital twins for type 2 diabetes

**DOI:** 10.3389/fdgth.2024.1336050

**Published:** 2024-01-26

**Authors:** Yue Zhang, Guangrong Qin, Boris Aguilar, Noa Rappaport, James T. Yurkovich, Lance Pflieger, Sui Huang, Leroy Hood, Ilya Shmulevich

**Affiliations:** ^1^ Institute for Systems Biology, Seattle, WA, United States; ^2^ Center for Phenomic Health, Buck Institute for Research on Aging, Novato, CA, United States; ^3^ Phenome Health, Seattle, WA, United States

**Keywords:** digital twin, type 2 diabetes, knowledge graph, machine learning, precision medicine

## Abstract

**Introduction:**

A digital twin is a virtual representation of a patient's disease, facilitating real-time monitoring, analysis, and simulation. This enables the prediction of disease progression, optimization of care delivery, and improvement of outcomes.

**Methods:**

Here, we introduce a digital twin framework for type 2 diabetes (T2D) that integrates machine learning with multiomic data, knowledge graphs, and mechanistic models. By analyzing a substantial multiomic and clinical dataset, we constructed predictive machine learning models to forecast disease progression. Furthermore, knowledge graphs were employed to elucidate and contextualize multiomic–disease relationships.

**Results and discussion:**

Our findings not only reaffirm known targetable disease components but also spotlight novel ones, unveiled through this integrated approach. The versatile components presented in this study can be incorporated into a digital twin system, enhancing our grasp of diseases and propelling the advancement of precision medicine.

## 1 Introduction

The concept of digital twins (DTs) has recently garnered attention in the realms of biomedical and clinical research, as well as among the general public. DTs were originally employed in the aerospace industry and subsequently in manufacturing and product life-cycle management (1, 2). In the biomedical field, the recognition that individual pathophysiological idiosyncrasies limit the applicability of traditional cohort-derived care guidelines has underscored the need for personalized (patient-tailored) disease management. In this evolving paradigm of clinical thinking—and buoyed by the surge in patient data made possible by molecular profiling technologies—the DT has emerged as a promising tool for realizing such highly personalized monitoring and intervention. A primary objective of a DT is to represent and simulate a patient's health trajectory, monitor disease progression, and discern potential treatment responses to guide corrective intervention. Given the inherent complexity of biological systems, DT can be designed with varying levels of abstraction and granularity, tailored to specific applications.

Within various disease domains, the concept of DTs has evolved into diverse and specialized variants. Hernandez-Boussard et al. proposed cancer patient digital twins that utilize emerging computing and biotechnologies to build *in silico* representations of individuals. These representations dynamically capture molecular, physiological, and lifestyle status across different treatment regimens and timelines in order to aid in clinical decision making (3). Voigt et al. suggested digital twins for multiple sclerosis that use artificial intelligence-based analysis of several disease parameters—including clinical and para-clinical outcomes, multiomics, biomarkers, patient-related data, and information about the patient's life circumstances and plans—as well as medical procedures, all paired to patient characteristics (4). Corral-Acero et al. emphasized the synergies between mechanistic and statistical models in cardiovascular digital twins (5). However, while existing disease-specific DTs have shown promise in enhancing our understanding and management of complex medical conditions, there remain critical gaps in the integration, scalability, and standardization of digital twin methodologies across various clinical applications, notably, in the ability to accommodate data derived from longitudinal monitoring.

Type 2 diabetes (T2D) is a disease with a complex and heterogeneous developmental process (6), making it an ideal candidate for evaluating the application of biomedical DTs. T2D is a metabolic disorder characterized by insulin resistance and, in later stages, relative insulin deficiency due to the exhaustion of pancreatic β-cells. It constitutes a growing global health crisis, with approximately 415 million people affected worldwide (7, 8). T2D also poses a high risk for cardiovascular diseases and chronic kidney disease (CKD), among other complications (9); it accounts for $1 of every $4 spent on healthcare in the United States (10). Given the increasing prevalence of T2D and the variability in its progression and prognosis across patient populations, the management of T2D stands to benefit from modeling through a DT system.

The extensive scientific understanding of the pathophysiology of T2D can be encoded into a knowledge graph, facilitating the organization and computational processing of complex information essential for constructing the DT. Knowledge graphs serve as expansive networks that map known relationships between various biomedical entities, such as genes, proteins, metabolites, drugs, and clinical phenotypes. These relationships may be derived from experiments, scientific literature, or ontological frameworks (11, 12). Knowledge graphs can contribute to both data analysis and modeling efforts. For example, they can assist in hypothesizing or explaining why certain entities constitute sets of features with predictive value. Alternatively, knowledge graphs can inform the development of predictive mechanistic models by providing curated causal relationships between entities. One of the most extensive biomedical knowledge graphs is SPOKE, “Scalable Precision Medicine Open Knowledge Engine,” a manually curated resource that encompasses over 27 million nodes and 53 million edges (13). The SPOKE knowledge graph consolidates information from 41 databases across diverse domains, including mechanistic information relevant to T2D.

**Figure 1:**
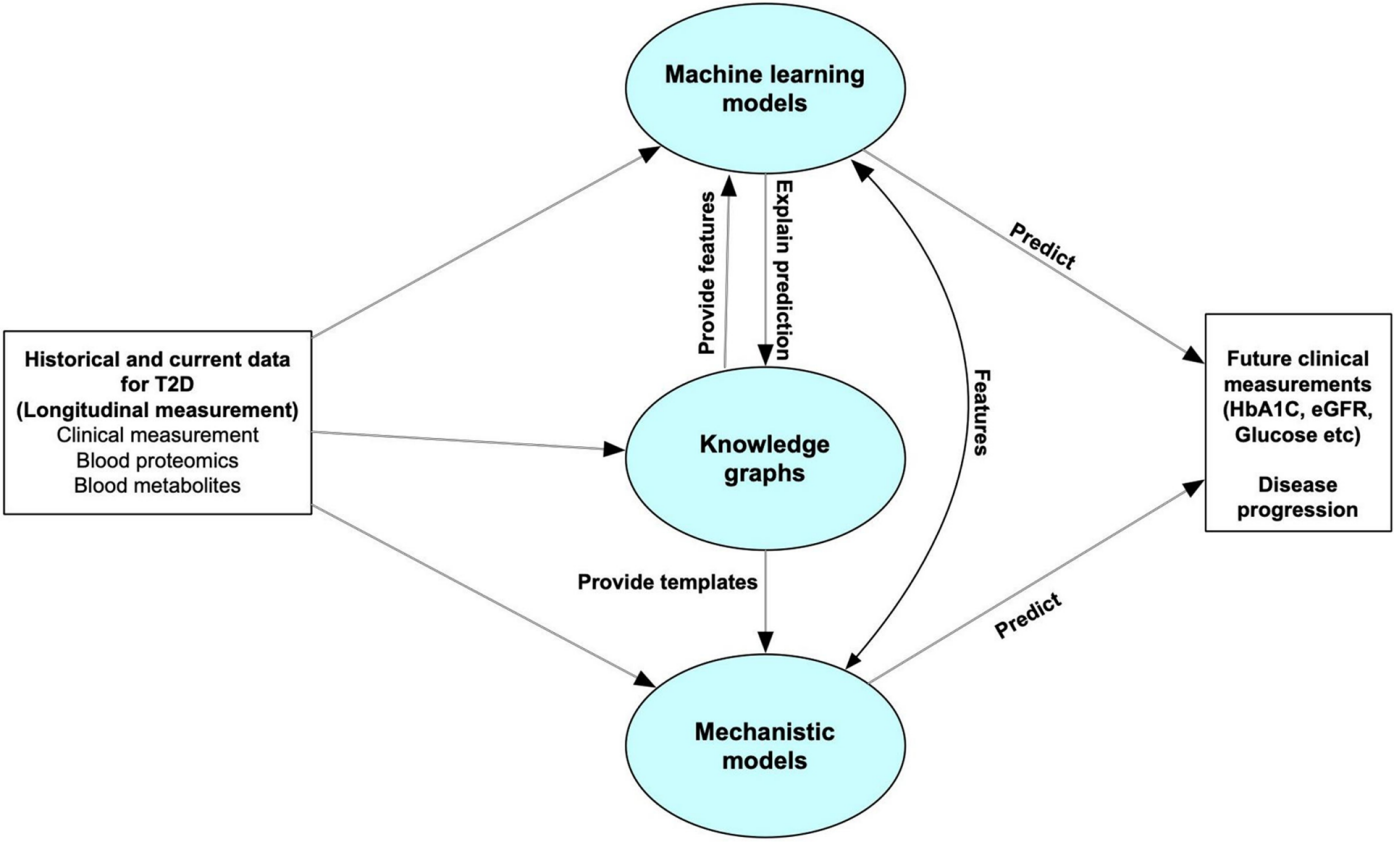
Overall structure of the digital twin system. The predictive core in our digital twin system includes machine learning models, knowledge graphs and mechanistic models. The machine learning models are used to forecast disease progression by predicting some clinical measurements which can be used for monitoring disease progression. Knowledge graphs can help select features for the machine learning models, and explain the predictive features. It can also leverage the personalized data to provide templates for mechanistic models. The Mechanistic models can give feedback to the machine learning models to improve the accuracy, or directly predict future clinical features or disease progression.

Here, we introduce a framework for a T2D DT designed to continuously monitor patients, assimilate high-dimensional (“omics scale”) data, and predict changes in clinical variables. Our DT comprises three key computational components: machine learning, knowledge graphs, and mechanistic models—the overall framework is illustrated in Figure 1. The machine learning models are trained to forecast disease progression and identify relevant clinical measurements for ongoing monitoring of disease progression, exemplified by variables such as glycated hemoglobin percentage (HbA1c) and estimated glomerular filtration rate (eGFR). Knowledge graphs, which encode existing scientific knowledge into a machine-readable format, can elucidate the predictive features identified by the machine learning models in terms of known mechanistic or causal relationships. They also assist in feature selection relevant to disease progression. Mechanistic models can serve as digital representations of individual patients but require a more comprehensive understanding of both the disease and the patient than current pathway knowledge provides. Therefore, the current study focuses primarily on the machine learning and knowledge graph elements of our designed digital twin system.

## 2 Materials and methods

### 2.1 Dataset description and processing

In this study we used the Arivale dataset, described in detail in (14, 15). Briefly, the Arivale dataset includes longitudinal data from ∼5,000 deeply phenotyped individuals undergoing a wellness program. These data include comprehensive self-reported data coupled with multiomic data (proteomics, metabolomics, clinical labs). The process of collecting multiomic and clinical data in Arivale has been described in the existing literature (15).

We selected a number of clinical variables related to T2D for further study: HbA1c (glycated hemoglobin percentage), eGFR (estimated glomerular filtration rate), glucose, insulin, and HOMA-IR (homeostatic model assessment of insulin resistance). HbA1c, Glucose, and Insulin are key clinical variables used to evaluate T2D progression in the clinical settings (16). We also included eGFR because we were interested in the transition from T2D to T2D with chronic kidney disease (CKD) as a complication (17). In order to train predictive models for changes in T2D-related clinical variables, after data pre-processing (see Methods), we selected a longitudinal dataset from 1,356 participants with data at ∼6 months, of which 738 had a longitudinal followup data at ∼1 year. In total, we analyzed 1,042 blood multiomic features: 262 proteins, 710 metabolites, and 70 clinical labs or demographic variables. A full list of variables is shown in Supplementary Table 1. To model the change in clinical variables over time, we computed the deltas (i.e., changes) in the selected clinical outcomes between the six-month and one-year follow-ups and the baseline (intake) values. These values are shown in Table 1.

**Table 1:** Table of sample characteristics. This table shows descriptive statistics for the test sample after filtering the data.

Variable	Count	Min	Mean	Median	Max	Std
Age	1,131	18.00	49.53	49.00	87.00	11.29
bmi	1,131	17.74	27.63	26.15	53.35	6.04
HbA1c	1,131	3.60	5.51	5.50	8.30	0.43
Glucose	1,131	70.00	93.24	91.00	199.00	12.18
eGFR	1,131	41.00	90.17	90.00	131.00	15.17
Insulin	1,131	1.40	10.67	8.90	55.10	7.34
HOMA-IR	1,131	0.25	2.56	1.98	20.34	2.16

Prior to analysis, the data were filtered and cleaned in a number of ways. First, we only included the subjects for which demographic, clinical, proteomic, and metabolomic data were available for at least two time points (*N* = 2,008). Next, we excluded subjects that had missing values for any of the five studied clinical variables in the first two time points. We then removed the clinical, proteomic, and metabolomic features that had more than 10% missingness across the data, and afterwards, we removed subjects with more than 10% missingness in the remaining metabolomic features, or any missingness in the remaining proteomic or clinical features. Imputation with missforest (18) (implemented in the python missingpy package) was performed to fill in missing values for the remaining metabolomic features. Subjects with values for clinical variables above certain thresholds (HbA1c>12, glucose >200, insulin >60) were removed. In total, this resulted in 1,131 subjects with data at 6 months, of which 639 had results at 1 year.

### 2.2 Machine learning models

An important component of a digital twin system is the implementation of predictive models that, given current and historical data, predicts the system's future state. To implement this aspect of the digital twin, we constructed machine learning models to predict the changes in T2D-related clinical outcome variables using clinical and multiomic data.

**Figure 2:**
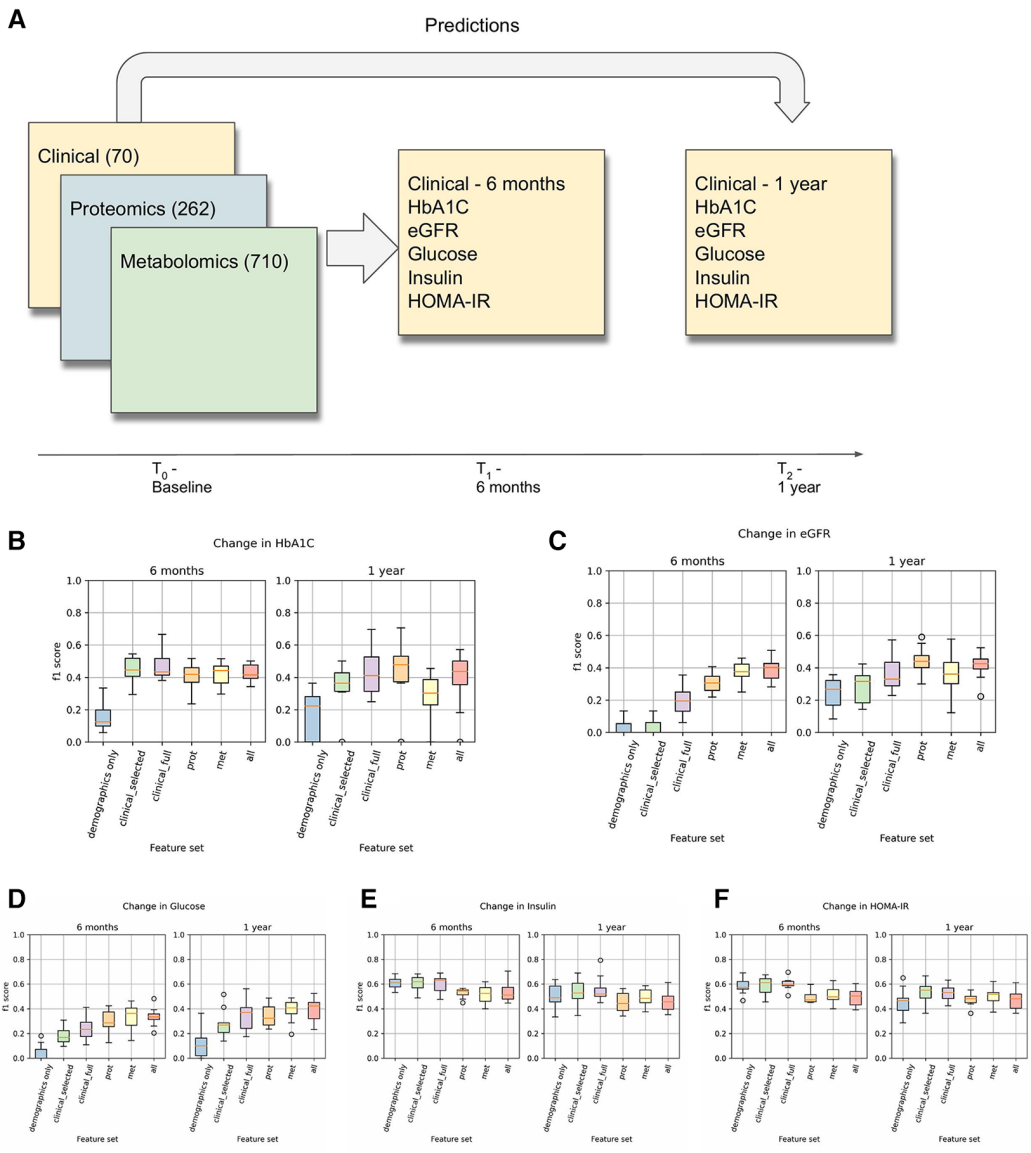
(**A**) This is an overview of the machine learning prediction method. Data from the intake time point were used to predict the values of the listed T2D-related clinical variables at 6 months and 1 year. (**B–F**) Prediction results for changes in HbA1c, eGFR, Glucose, Insulin, and HOMA-IR. These results used a logistic regression model (for classification) with L1 regularization on each of the listed feature sets to predict whether the given clinical variable would increase by at least 5% (or decrease by at least 5% in the case of eGFR).

The overall process of predicting clinical trajectories is described in Figure 2A. Using the datasets described in Table 2, including clinical, proteomic, and metabolomic features, we trained machine learning models to predict the changes (delta) in the values of the five key T2D-related clinical variables (HbA1c, glucose, insulin, HOMA-IR, eGFR). Since values for the selected clinical tests generally remain steady over time, we used the baseline test results as an additional feature when predicting the deltas. Using data from time t0 (the first recorded data for a given subject), we predicted the changes at times t1 (approximately 6 months) and t2 (approximately 1 year). In every case, we used 10-fold cross-validation to assess the performance of the models and feature sets, using 90% of the data for training and 10% of the data for testing. *Z*-score normalization was used on all of the feature sets, applying the mean and variance for each variable from the training set on both the training and test sets. Machine learning models were implemented using the Scikit-learn library in python (19).

**Table 2:** Description of the feature sets used in predicting clinical trajectories. All feature sets include the baseline feature as one of the variables.

Feature set	# Variables	Description
Baseline	1	Only using the t0 value of the clinical variable being predicted.
Demographics only	4	Age, sex, BMI (+ baseline)
Clinical—selected	15	Selected clinical + demographic variables associated with T2D
Clinical—full	71	All clinical + demographic variables (with less than 10% missingness threshold)
Proteomics	263	All proteins with less than 10% missingness
Metabolomics	711	All metabolites with less than 10% missingness
All	1,043	All of the above combined

We used both regression and classification models for the delta predictions. In regression, we predicted the value of the change. To convert this into a classification problem, we designed a binary classification task where a positive value indicates an increase of at least 5% in the clinical variable from t0 to t1 or t2 (or a 5% decrease in the case of eGFR). Binary variables were generated for all five clinical variables at both time points. We hypothesized that transforming the prediction task into a classification problem would render the analysis more robust against the fluctuations observed over the relatively short follow-up time scales. A description of the changes in the clinical variables is shown in Table 3.

**Table 3:** Table of changes in the clinical variables at 6 months and 1 year. The “# >= 5%” column indicates the number of samples that had an increase (or decrease in the case of eGFR) in the variable of at least 5%.

Changes after 6 months							
	Count	Min	Mean	Median	Max	Std	# >= 5%
d_HbA1c	1,131	−1.50	−0.05	−0.10	1.40	0.29	185
d_Glucose	1,131	−49.00	−0.48	0.00	39.00	7.78	299
d_GFR	1,131	−30.00	1.04	0.00	45.00	9.44	250
d_Insulin	1,131	−31.50	−0.89	−0.40	22.70	4.97	453
d_HOMA-IR	1,131	−11.09	−0.24	−0.12	7.09	1.43	455
Changes after 1 year							
d_1y_HbA1c	639	−1.90	−0.11	−0.10	1.00	0.31	87
d_1y_Glucose	639	−57.00	−0.18	1.00	51.00	8.89	204
d_1y_GFR	639	−28.00	−0.05	0.00	53.00	10.44	176
d_1y_Insulin	639	−33.20	−0.98	−0.60	54.40	5.41	247
d_1y_HOMA-IR	639	−12.90	−0.25	−0.11	23.29	1.80	253

We tested a variety of machine learning models for these tasks, including various linear models as well as nonlinear models for regression and classification such as random forests and support vector machines. All of the regression models and classification models are shown in Supplementary Table 4.

### 2.3 Graph analysis

Considering the importance of interpretability in healthcare digital twins and the ability to select highly predictive features based on regression model weights, we aimed to discern the relationships among these highly weighted features and their connection to the disease processes in T2D and CKD. To accomplish this, we leveraged the SPOKE knowledge graph (13). Our version of this graph consists of approximately 2 million nodes (with 22 types) and over 14 million edges (54 types); a description of the graph's nodes and edges is in Supplementary Table 3. Knowledge graphs such as SPOKE can be used to explore the relationships among the most predictive features, generate hypotheses regarding the connections between these features and the disease processes in T2D and CKD, and identify potentially significant yet unmeasured related features (genes, proteins, metabolites) for subsequent investigation.

In our knowledge graph analyses, we applied Steiner tree approximation (20) and topic PageRank (21) algorithms. We used the python-igraph package for all of our knowledge graph work (22), as well as some custom implementations of Steiner tree approximation methods based on the Takahashi method described in (20).

## 3 Results

### 3.1 Predicting clinical trajectories

As described in the Methods section, we trained various classification and regression machine learning models to predict the clinical trajectory of T2D-related variables. Results and metrics for all of the tasks are shown in Supplementary Table 2. Comparing the results across different models, we found that LassoCV models for regression and L1-regularized logistic regression models for classification tended to give the best predictions. In most cases, treating the prediction as a binary classification problem led to performance results that had lower variance across the cross-validation runs, especially at 1 year, and a clearer differentiation in the performance between the different feature sets.

Results for the classification-based predictions are shown in Figures 2B–E. This shows the F1 score of the L1-regularized logistic regression classifier, with the F1 scores calculated on the held-out test sets in a 10-fold cross-validation. From this, we observed that the full clinical, proteomic, and metabolomic feature sets all performed better than the demographics-only feature set for predicting changes in HbA1C, Glucose, and eGFR at 6 months and 1 year. We also observed that the proteomic and metabolomic feature sets performed better than the clinical feature sets for predictions of eGFR and Glucose at 6 months, illustrating the utility of multiomic data. However, the performance of the Insulin and HOMA-IR predictors were approximately the same regardless of the feature set. In the Arivale dataset, insulin levels tended to fluctuate more than the other clinical variables (Table 3), and there appears to be a regression-to-the-mean effect where high baseline insulin values tend to lead to a future decrease (Supplementary Figure 4).

Our results highlight the application of multiomic data in predicting T2D trajectories. Previously, multiomics have been used to predict the progression of T2D, with some success (23–26). Only the work by Prélot et al. (23) predicts clinical variables (including fasting glucose, fasting insulin, and HbA1c) rather than predicting T2D status, but the set of metabolites used are mostly considered to be clinical variables in our study. In that study, fasting insulin had the most accurate prediction at a 15-year follow-up using metabolite features (with an *R*^2^ of 0.54), while HbA1c was not able to be accurately predicted (with only a 0.15 *R*^2^ value). Our results here use much shorter time scales than the previous studies—less than one year vs. multiple years to decades. This could lead to more noise, as the values of clinical variables often fluctuate. However, despite the noise, significant predictions of short-term changes were possible with clinical, proteomic, and metabolomic data.

### 3.2 Exploring predictive models using knowledge graphs

While predictive models are one key component of a digital twin, another key component is the interpretability of the predictions. The linear models we used provide weights for all features, and the highest-weighted features for the dHbA1c and deGFR (delta-HbA1c and delta-eGFR) 6-month predictors are shown in Supplementary Figures 1,2. However, in a high-dimensional multiomic setting, it might be difficult to understand the significance of individual features. Leveraging the knowledge graph's rich connections might aid us in understanding.

**Figure 3:**
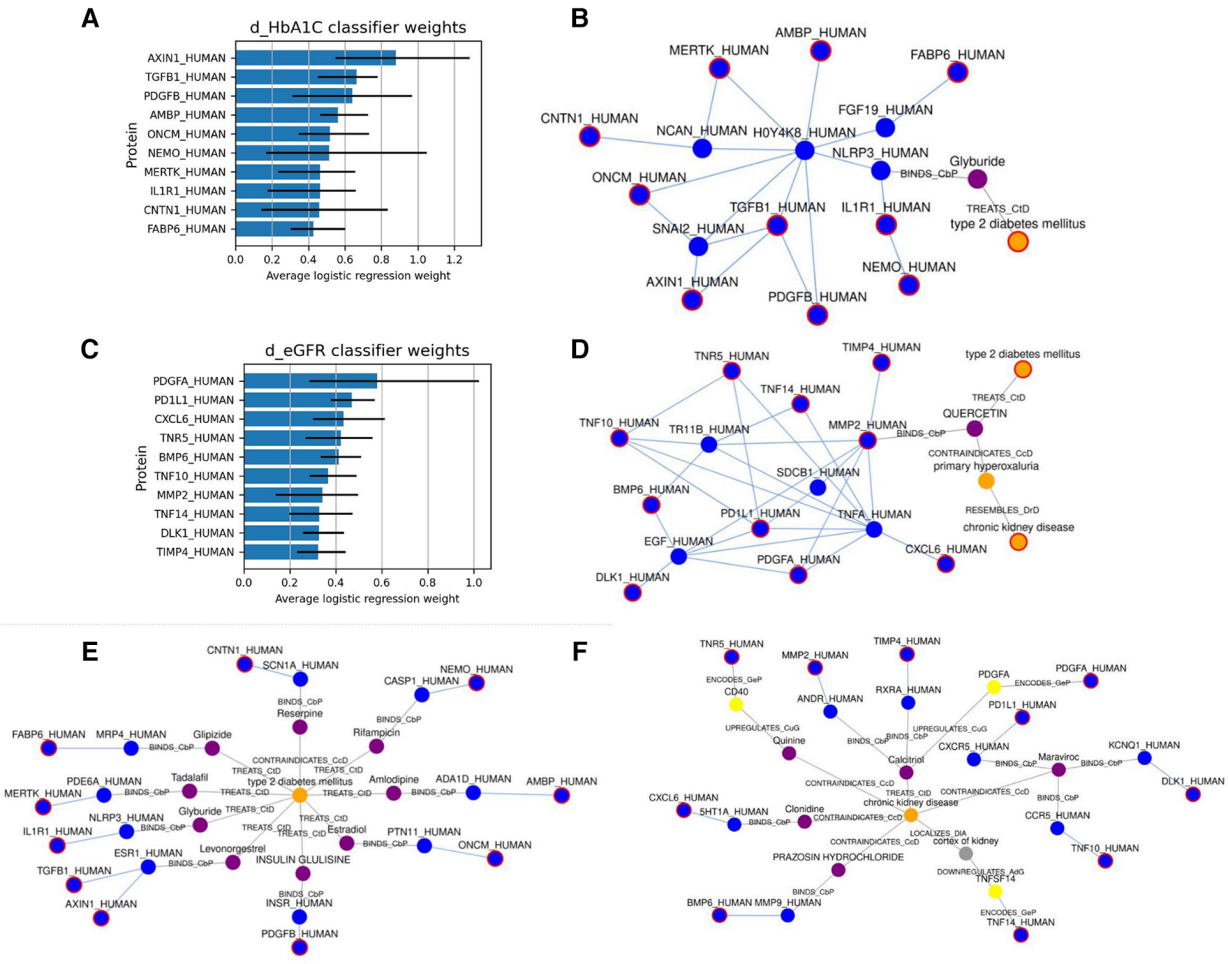
(**A,C**) show the top 10 highest weighted protein predictors for dHbA1c and deGFR, respectively, at 6 months, using L1-regularized logistic regression. The bars indicate the range that the coefficients take over the 10 cross-validation runs. (**B,D**) Show the approximate Steiner subgraph on SPOKE using the top 10 highest weighted proteins. A node outlined in red is part of the input set. Blue nodes are proteins, while orange nodes are diseases, and purple nodes are compounds. Light blue edges represent protein-protein interactions, while gray edges represent all other types of connections, and are labeled with the edge type. (**E,F**) Show the shortest paths from each of the top 10 proteins to the nodes for T2D and CKD.

First, we used SPOKE (13) to identify relationships between the highly predictive features and T2D. Based on the Network Parsimony principle (27), we identify the shortest paths from each of the predictor protein nodes to the T2D node, which might represent molecular pathways of action. The graph for the dHbA1c predictors is shown in Figure 3E, while the graph for the deGFR predictors is shown in Figure 3F. Examining the shortest paths to T2D, we see that the paths run through many known drugs for treating T2D, such as glipizide and glyburide (8), and more proteins that are connected to both these drugs and the predictive proteins. In a personalized medicine setting, this could potentially identify treatment paths.

We also identified a subgraph of related nodes consisting of the top predictive features (here, they are proteins mapped to nodes in SPOKE), as well as the disease node for Type 2 Diabetes. We constructed a subgraph using an approximate Steiner tree approach described in (20). Expanding the Steiner tree to include all edges within the tree's nodes gives us the (approximately) smallest subgraph that contains all of the nodes of interest. These results are shown in Figures 3B,D for the dHbA1c and deGFR predictors. This shows both the relationships within the feature sets as well as one possible relationship with T2D. Using knowledge graphs allows us to explore indirect connections among the top predictive features, and between features and the disease of interest. We can see that the top predictive features are densely connected by protein-protein interaction edges, and we can see how some nodes are connected to T2D. Since the proteomic data only included hundreds of proteins, there are many additional proteins that could be predictive of a T2D trajectory, but have not been measured, and identifying proteins that are highly connected to measured predictive proteins could suggest new features to measure. For example, NLRP3, which connects the predictive proteins to the T2D node, is known to be related to T2D progression (28, 29). In the deGFR predictors subgraph, TNFA is connected to many predictive proteins and is also known to be related to the progression of CKD (30). This indicates using the knowledge graphs, we can not only find the relationship between the predictive features and disease, but also find additional features or genes that are relevant to the disease.

**Figure 4:**
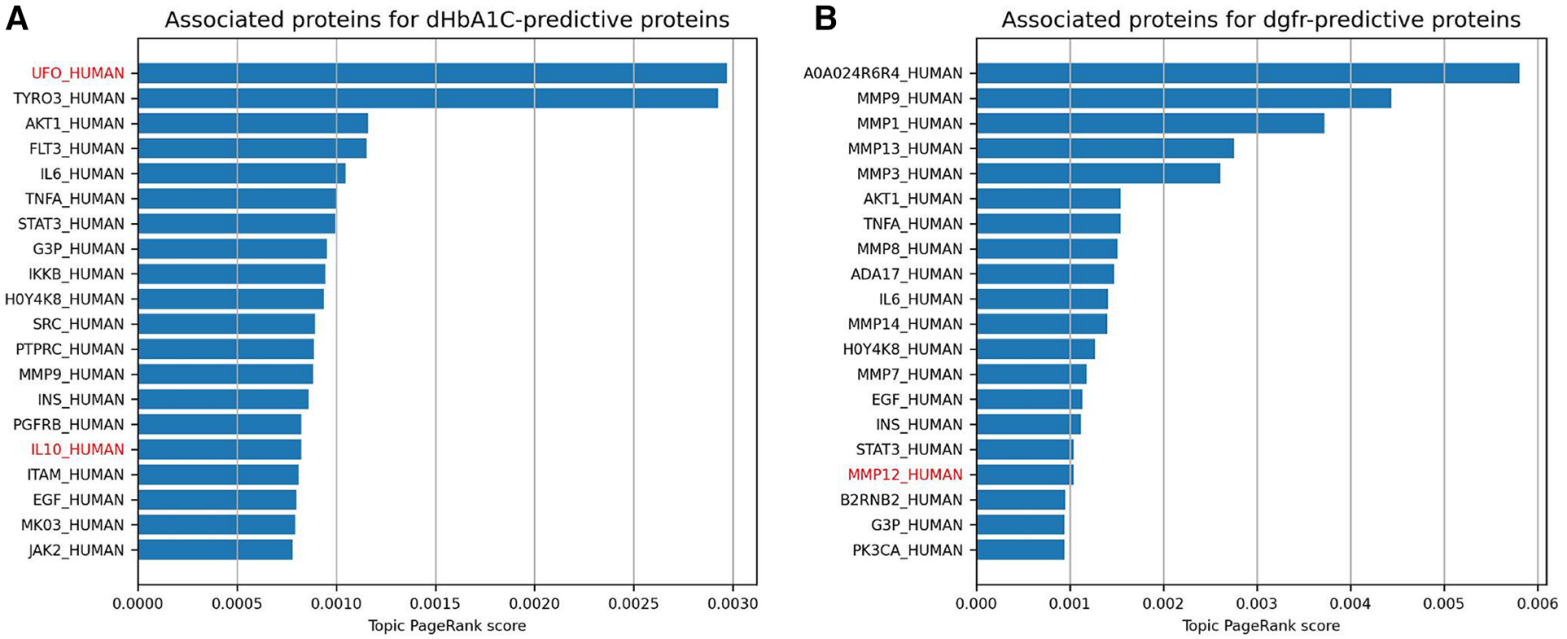
This shows the highest topic pageRank scores for protein nodes in SPOKE, using the top 10 highest weighted protein predictors for dHbA1c and deGFR, respectively. The query set of features are the same as the proteins in the figure above. Names with red text indicate proteins that also have positive weights for the given prediction task across all 10 cross-validation runs, but were not one of the 10 highest.

The topic PageRank algorithm is another way of identifying features on the graph that are related to the features in question, including potential features that are not currently being measured but might be of interest for future study (31, 21). Topic PageRank computes a random walk with restarts, where the restarts will return to a set of query nodes, and returns as a weight the probability of landing in each node in the graph. This algorithm has been previously applied to gene prioritization, identifying key genes for a disease by finding indirect associations from a set of seed genes (32). This is essentially what we are doing here, using highly predictive proteins as seed nodes. Figures 4A,B show the protein nodes with the highest topic PageRank scores when querying SPOKE with the top 10 highest-weighted protein predictors for dHbA1c and deGFR, respectively. The proteins highlighted in red, UFO and IL10, are not in the top 10 but are also highly weighted predictors for the same target in the logistic regression model, with nonzero weights across all cross-validation runs. This indicates that the topic PageRank method is able to retrieve features that are known to be relevant to the clinical target. As with the Steiner tree-identified additional proteins, some of the additional topic PageRank-identified proteins that are not measured in this study have been shown to be associated with T2D, including TYRO3 and AKT1 (33–35). For deGFR, most of the topic PageRank-identified proteins are part of the MMP (matrix metalloproteinase) family, which have been shown to have associations with chronic kidney disease (36, 37).

## 4 Discussion

In our digital twin system design, which incorporates machine learning, knowledge graphs and mechanistic models, we have demonstrated the utility of integrating machine learning with knowledge graphs for predicting disease trajectories and identifying key features as well as their relationships to the disease. While digital twins have been applied extensively in various engineering disciplines, their application to biomedical research has been limited. Their limited application is primarily attributed to the inherent complexity of biological systems, which poses a unique challenge in their modeling and simulation. Here, we attempt to overcome this challenge by integrating extensive mechanistic information represented in an existing biomedical knowledge graph with deep phenotyping data to model T2D. Through our work, we have identified proteomic, metabolomic, and clinical features that can be used to predict changes in a number of clinical variables related to T2D over the course of 6 months to 1 year. Our results suggested proteomic and metabolomic features can provide a better prediction than clinical features for some clinical tests. Our approach of integration of the ML models with the knowledge graph highlighted known disease targets and potential interventions.

Our results have two key implications. First, the improved predictive power that comes from integrating multiomic data with clinical features suggests that omic data could have added value as part of standard clinical care. Zubair et al. showed that on average, measures of metabolic health improved over the course of the Arivale study, with a significant reduction in HbA1c on average, due to the treatment and counseling provided (14). However, this masks significant heterogeneity in the outcomes. In fact, 374 and 167 subjects showed an increase in HbA1c over 6 months and 1 year, respectively, as opposed to 604 and 399 who showed a decrease. There are hurdles associated with translating omic technologies to clinical use—including cost, technology development, and education (38)—but our results show that their inclusion increases predictive value. Other studies have shown that omic data integrated with clinical labs can yield increased model performance, but these results are for health metrics (15) and not clinically validated tests. Second, the ability to interpret model predictions via a knowledge graph provides a robust framework for the implementation of biomedical digital twins. We have demonstrated that utilizing knowledge graphs and phenomic data can interpret predicted features, both validating known pathophysiology and predicting novel disease targets.

One of the limitations encountered is the sparsity of longitudinal data. Ideally, a system such as the one proposed here would be validated using densely sampled time courses, offering a high-resolution view into the dynamics of the disease and continuously updating the DT with this information. However, a densely sampled longitudinal phenomic dataset does not yet exist; thus, we have addressed these limitations to the best of our ability. Moreover, the limited sample size of the study complicates the construction of predictive models, particularly for high-dimensional multiomic data. Additionally, since the data predominantly originated from generally healthy participants, building models for progression specifically within a T2D disease state was not feasible. Incorporating more data specifically from participants with T2D would be valuable, but it would likely introduce patients with more comorbidities and could introduce confounders into the data that would need careful corrections.

The next steps for the framework proposed here is to move beyond purely statistical modeling by directly integrating mechanistic models into the pipeline. There has been a significant amount of work on mechanistic modeling for biological processes involved in T2D, but these models generally do not involve multiomic data and tend to be over shorter time scales (39). One way to approach this endeavor would be to use machine learning to determine model parameters for a mechanistic model (40), an approach that has proven useful in the estimation of kinetic parameters in bacteria (41). Further improvements would include a dashboard and user interface that would enable a broader adoption of DTs, possibly with natural language interfaces employing large language models to interpret and return results (42).

## Data Availability

The data analyzed in this study is subject to the following licenses/restrictions: The Arivale dataset is available upon request. Requests to access these datasets should be directed to data-access@isbscience.org. The SPOKE knowledge graph used in this study was provided by https://spoke.ucsf.edu/. Code used to analyze the data is available at https://github.com/IlyaLab/t2d-dt-modeling. Additional code for knowledge graph analysis is available at https://github.com/yjzhang/kg_feature_engineering.

## Supplementary material

Supplementary Figure 1, Supplementary Figure 2, Supplementary Figure 3, Supplementary Table 4 All nonzero coefficients for the dHbA1c logistic regression predictors at 6 months, using the clinical, proteomic, and metabolomic feature sets. The bars indicate the range of coefficient values across all cross-validation runs. All nonzero coefficients for the deGFR logistic regression predictors at 6 months, using the clinical, proteomic, and metabolomic feature sets. The bars indicate the range of coefficient values across all cross-validation runs. Prediction results for changes in HbA1c, eGFR, Glucose, Insulin, and HOMA-IR, using a LASSO regression model for predicting the delta values. All of the machine learning models used for predicting changes in clinical variables.

Supplementary Table 1 List of all multiomic features used for the different prediction models.

Supplementary Table 2 Tables showing all prediction results for the multiomic models, with R^2^ metrics for the regressions and F1 metrics for the classifications.

Supplementary Table 3 An overview of the nodes and edges of the SPOKE knowledge graph.
