# Supplementary material for: A framework towards digital twins for type 2 diabetes
Source: Front Digit Health. 2024 Jan 26;6:1336050. doi: 10.3389/fdgth.2024.1336050 (PMC10853398; doi:10.3389/fdgth.2024.1336050)
Supplement: Supplementary Figure 1, Supplementary Figure 2, Supplementary Figure 3, Supplementary Table 4 — All nonzero coefficients for the dHbA1c logistic regression predictors at 6 months, using the clinical, proteomic, and metabolomic feature sets. The bars indicate the range of coefficient values across all cross-validation runs. All nonzero coefficients for the deGFR logistic regression predictors at 6 months, using the clinical, proteomic, and metabolomic feature sets. The bars indicate the range of coefficient values across all cross-validation runs. Prediction results for changes in HbA1c, eGFR, Glucose, Insulin, and HOMA-IR, using a LASSO regression model for predicting the delta values. All of the machine learning models used for predicting changes in clinical variables. [file Datasheet1.pdf]

Supplementary Material

1 Supplementary Data

Supplementary Material should be uploaded separately on submission. Please include any supplementary data, figures and/or tables.

Supplementary material is not typeset so please ensure that all information is clearly presented, the appropriate caption is included in the file and not in the manuscript, and that the style conforms to the rest of the article.

2 Supplementary Figures and Tables

For more information on Supplementary Material and for details on the different file types accepted, please see here.

2.1 Supplementary Figures

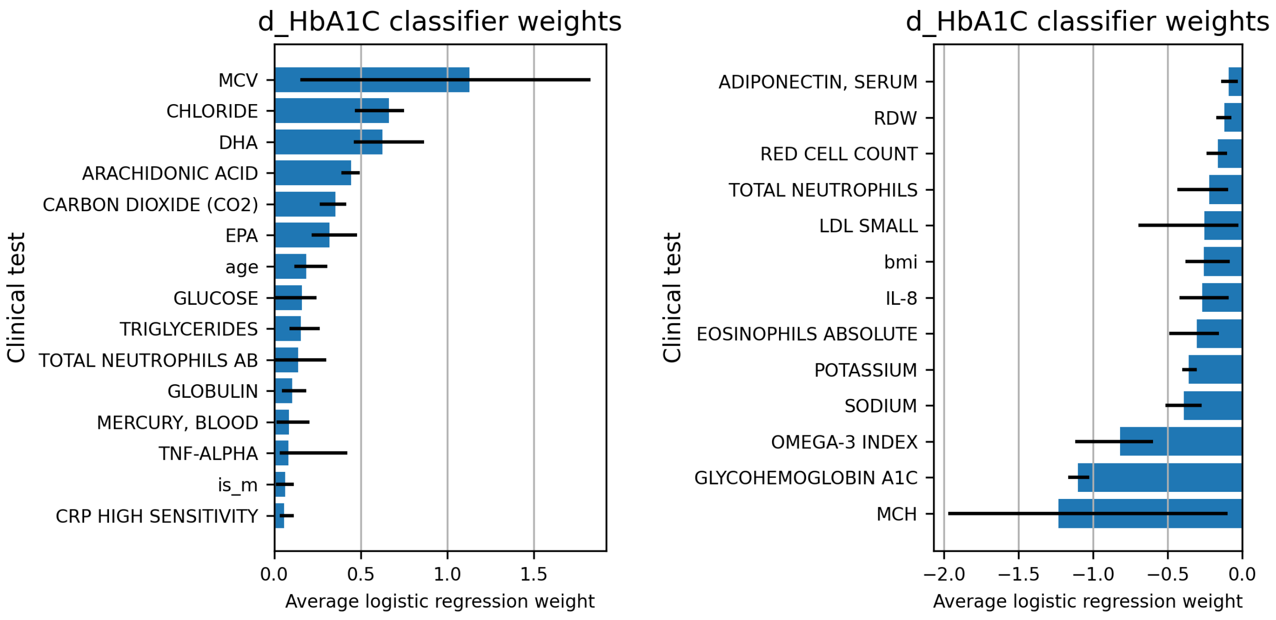

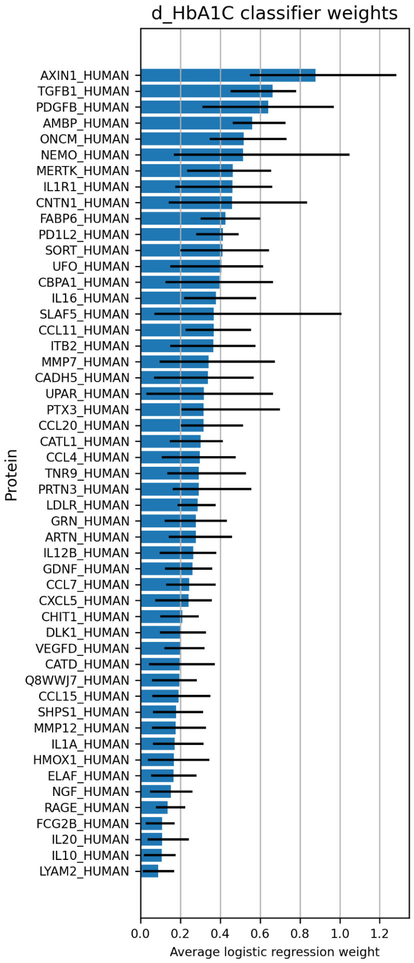
d_HbA1C classifier weights
AXIN1_HUMAN
TGFB1_HUMAN
PDGFB_HUMAN
AMBP_HUMAN
ONCM_HUMAN
NEMO_HUMAN
MERTK_HUMAN
IL1R1_HUMAN
CNTN1_HUMAN
FABP6_HUMAN
PD1L2_HUMAN
SORT_HUMAN
UFO_HUMAN
CBPA1_HUMAN
IL16_HUMAN
SLAF5_HUMAN
CCL11_HUMAN
ITB2_HUMAN
MMP7_HUMAN
CADH5_HUMAN
UPAR_HUMAN
PTX3_HUMAN
CCL20_HUMAN
CATL1_HUMAN
CCL4_HUMAN
TNR9_HUMAN
PRTN3_HUMAN
LDLR_HUMAN
GRN_HUMAN
ARTN_HUMAN
IL12B_HUMAN
GDNF_HUMAN
CCL7_HUMAN
CXCL5_HUMAN
CHIT1_HUMAN
DLK1_HUMAN
VEGFD_HUMAN
CATD_HUMAN
Q8WWJ7_HUMAN
CCL15_HUMAN
SHPS1_HUMAN
MMP12_HUMAN
IL1A_HUMAN
HMOX1_HUMAN
ELAF_HUMAN
NGF_HUMAN
RAGE_HUMAN
FCG2B_HUMAN
IL20_HUMAN
IL10_HUMAN
LYAM2_HUMAN
Protein
0.0
0.2
0.4
0.6
0.8
1.0
1.2
Average logistic regression weight

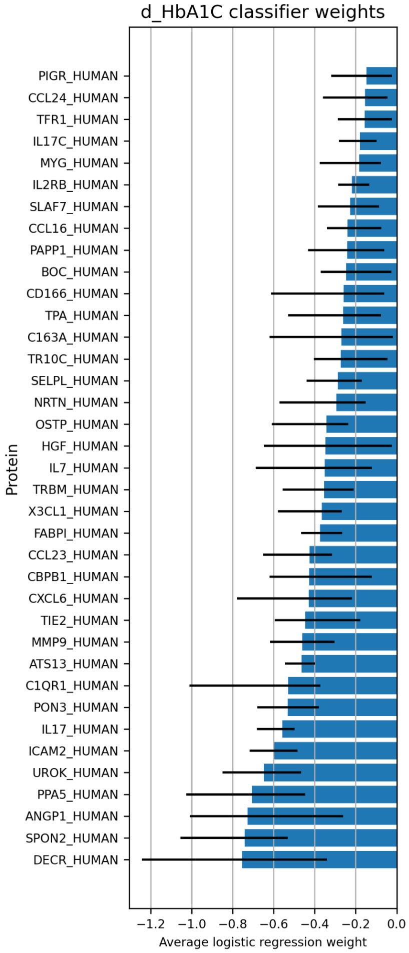
d_HbA1C classifier weights
PIGR_HUMAN
CCL24_HUMAN
TFR1_HUMAN
IL17C_HUMAN
MYG_HUMAN
IL2RB_HUMAN
SLAF7_HUMAN
CCL16_HUMAN
PAPP1_HUMAN
BOC_HUMAN
CD166_HUMAN
TPA_HUMAN
C163A_HUMAN
TR10C_HUMAN
SELPL_HUMAN
NRTN_HUMAN
OSTP_HUMAN
HGF_HUMAN
IL7_HUMAN
TRBM_HUMAN
X3CL1_HUMAN
FABPI_HUMAN
CCL23_HUMAN
CBPB1_HUMAN
CXCL6_HUMAN
TIE2_HUMAN
MMP9_HUMAN
ATS13_HUMAN
C1QR1_HUMAN
PON3_HUMAN
IL17_HUMAN
ICAM2_HUMAN
UROK_HUMAN
PPA5_HUMAN
ANGP1_HUMAN
SPON2_HUMAN
DECR_HUMAN
Protein
−1.2
−1.0
−0.8
−0.6
−0.4
−0.2
0.0
Average logistic regression weight

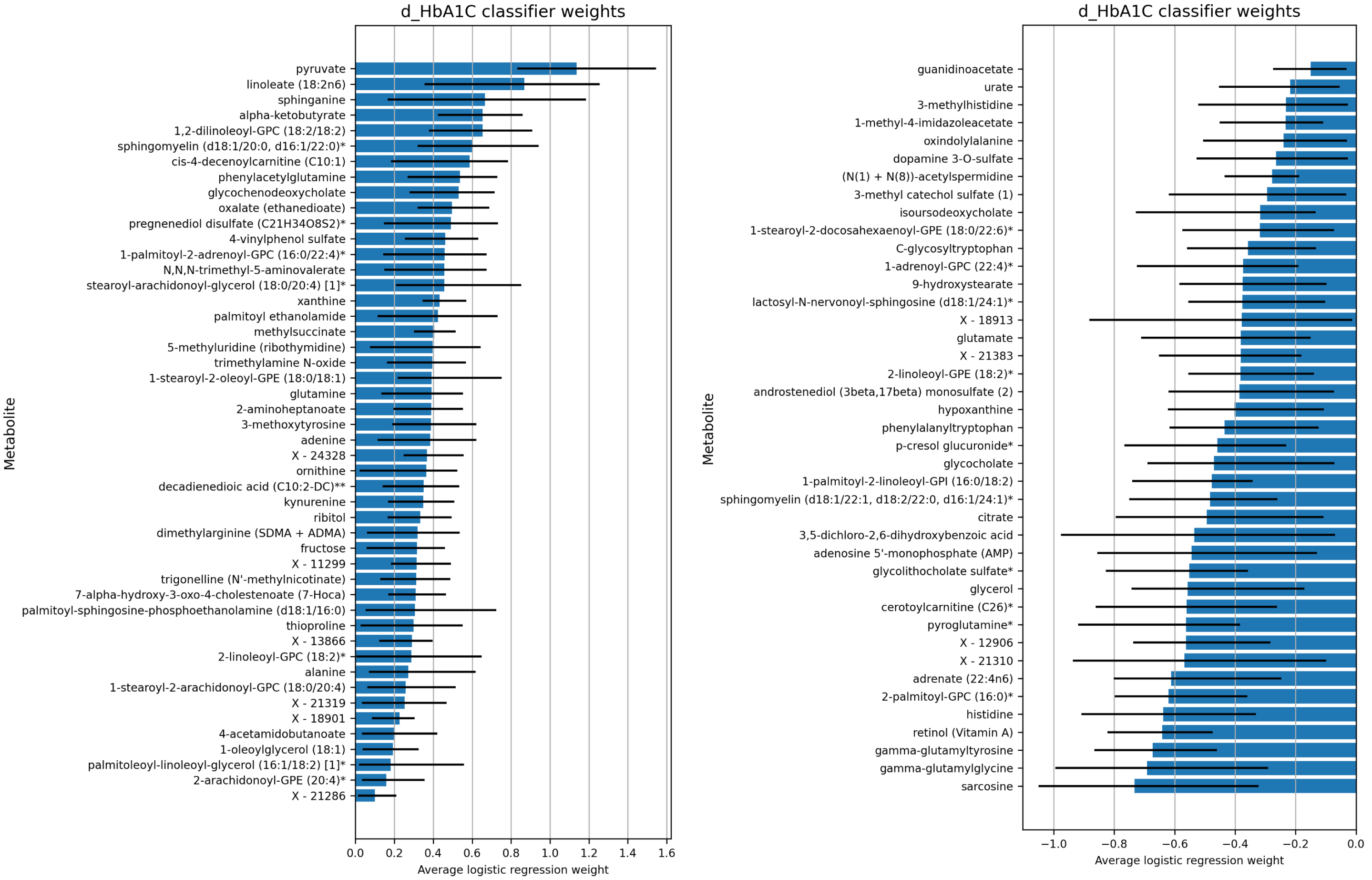

Supplementary Figure 1. All nonzero coefficients for the dHbA1C logistic regression predictors at 6 months, using the clinical, proteomic, and metabolomic feature sets. The bars indicate the range of coefficient values across all cross-validation runs.

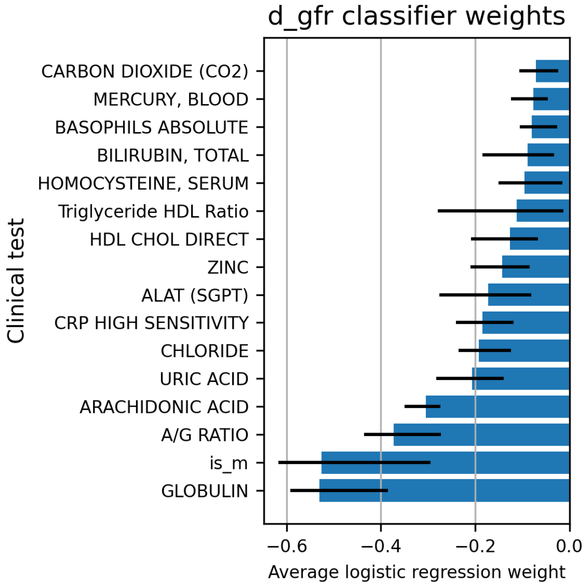
d_gfr classifier weights
CARBON DIOXIDE (CO2)
MERCURY, BLOOD
BASOPHILS ABSOLUTE
BILIRUBIN, TOTAL
HOMOCYSTEINE, SERUM
Triglyceride HDL Ratio
HDL CHOL DIRECT
ZINC
ALAT (SGPT)
CRP HIGH SENSITIVITY
CHLORIDE
URIC ACID
ARACHIDONIC ACID
A/G RATIO
is_m
GLOBULIN
Clinical test
−0.6
−0.4
−0.2
0.0
Average logistic regression weight

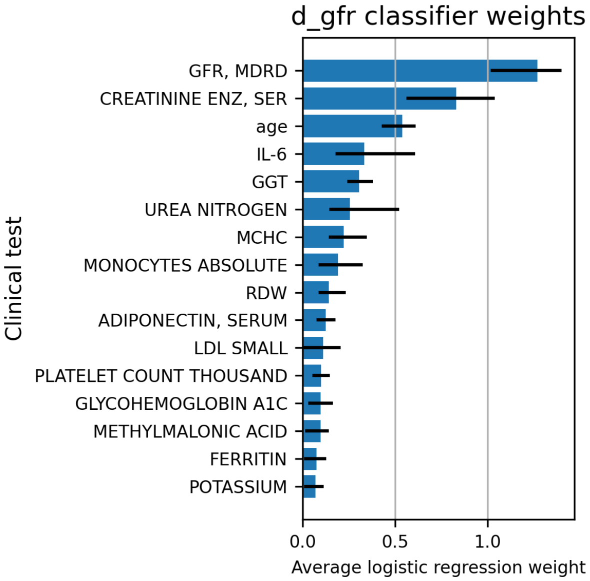
d_gfr classifier weights
GFR, MDRD
CREATININE ENZ, SER
age
IL-6
GGT
UREA NITROGEN
MCHC
MONOCYTES ABSOLUTE
RDW
ADIPONECTIN, SERUM
LDL SMALL
PLATELET COUNT THOUSAND
GLYCOHEMOGLOBIN A1C
METHYLMALONIC ACID
FERRITIN
POTASSIUM
Clinical test
0.0
0.5
1.0
Average logistic regression weight

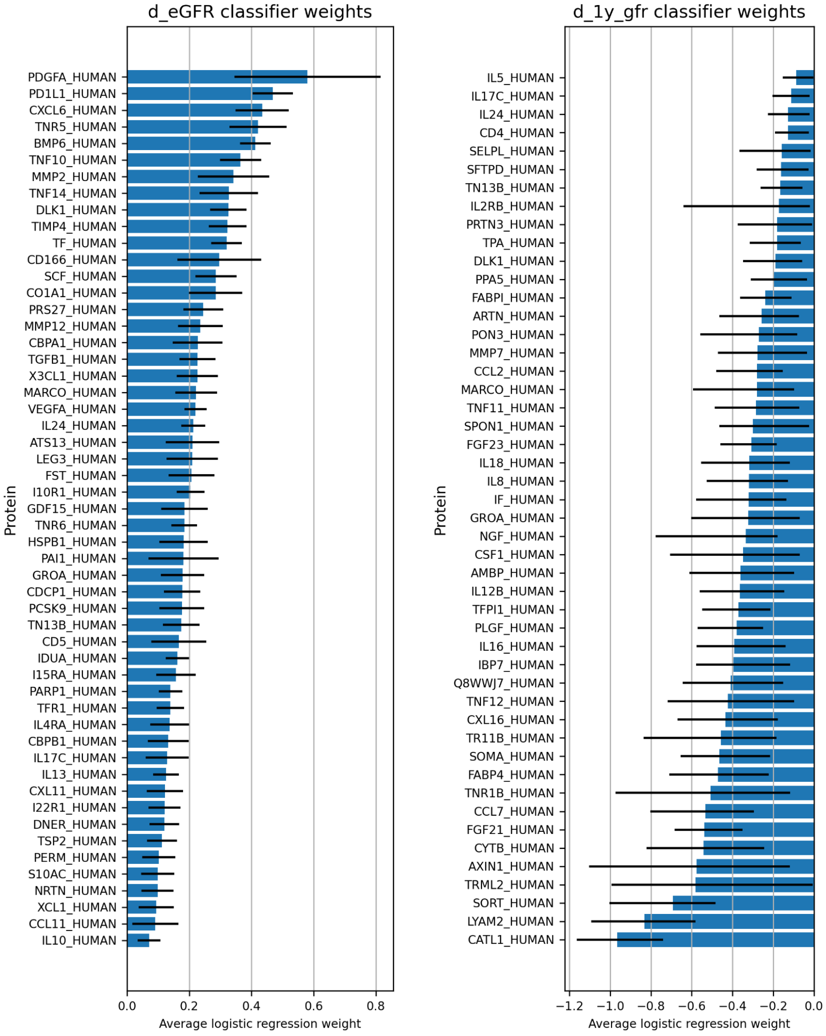

d_eGFR classifier weights
Protein
Average logistic regression weight
PDGFA_HUMAN
PD1L1_HUMAN
CXCL6_HUMAN
TNR5_HUMAN
BMP6_HUMAN
TNF10_HUMAN
MMP2_HUMAN
TNF14_HUMAN
DLK1_HUMAN
TIMP4_HUMAN
TF_HUMAN
CD166_HUMAN
SCF_HUMAN
CO1A1_HUMAN
PRS27_HUMAN
MMP12_HUMAN
CBPA1_HUMAN
TGFB1_HUMAN
X3CL1_HUMAN
MARCO_HUMAN
VEGFA_HUMAN
IL24_HUMAN
ATS13_HUMAN
LEG3_HUMAN
FST_HUMAN
I10R1_HUMAN
GDF15_HUMAN
TNR6_HUMAN
HSPB1_HUMAN
PAI1_HUMAN
GROA_HUMAN
CDCP1_HUMAN
PCSK9_HUMAN
TN13B_HUMAN
CD5_HUMAN
IDUA_HUMAN
I15RA_HUMAN
PARP1_HUMAN
TFR1_HUMAN
IL4RA_HUMAN
CBPB1_HUMAN
IL17C_HUMAN
IL13_HUMAN
CXL11_HUMAN
I22R1_HUMAN
DNER_HUMAN
TSP2_HUMAN
PERM_HUMAN
S10AC_HUMAN
NRTN_HUMAN
XCL1_HUMAN
CCL11_HUMAN
IL10_HUMAN
0.0
0.2
0.4
0.6
0.8
d_1y_gfr classifier weights
Protein
Average logistic regression weight
IL5_HUMAN
IL17C_HUMAN
IL24_HUMAN
CD4_HUMAN
SELPL_HUMAN
SFTPD_HUMAN
TN13B_HUMAN
IL2RB_HUMAN
PRTN3_HUMAN
TPA_HUMAN
DLK1_HUMAN
PPA5_HUMAN
FABPI_HUMAN
ARTN_HUMAN
PON3_HUMAN
MMP7_HUMAN
CCL2_HUMAN
MARCO_HUMAN
TNF11_HUMAN
SPON1_HUMAN
FGF23_HUMAN
IL18_HUMAN
IL8_HUMAN
IF_HUMAN
GROA_HUMAN
NGF_HUMAN
CSF1_HUMAN
AMBP_HUMAN
IL12B_HUMAN
TFPI1_HUMAN
PLGF_HUMAN
IL16_HUMAN
IBP7_HUMAN
Q8WWJ7_HUMAN
TNF12_HUMAN
CXL16_HUMAN
TR11B_HUMAN
SOMA_HUMAN
FABP4_HUMAN
TNR1B_HUMAN
CCL7_HUMAN
FGF21_HUMAN
CYTB_HUMAN
AXIN1_HUMAN
TRML2_HUMAN
SORT_HUMAN
LYAM2_HUMAN
CATL1_HUMAN
−1.2
−1.0
−0.8
−0.6
−0.4
−0.2
0.0

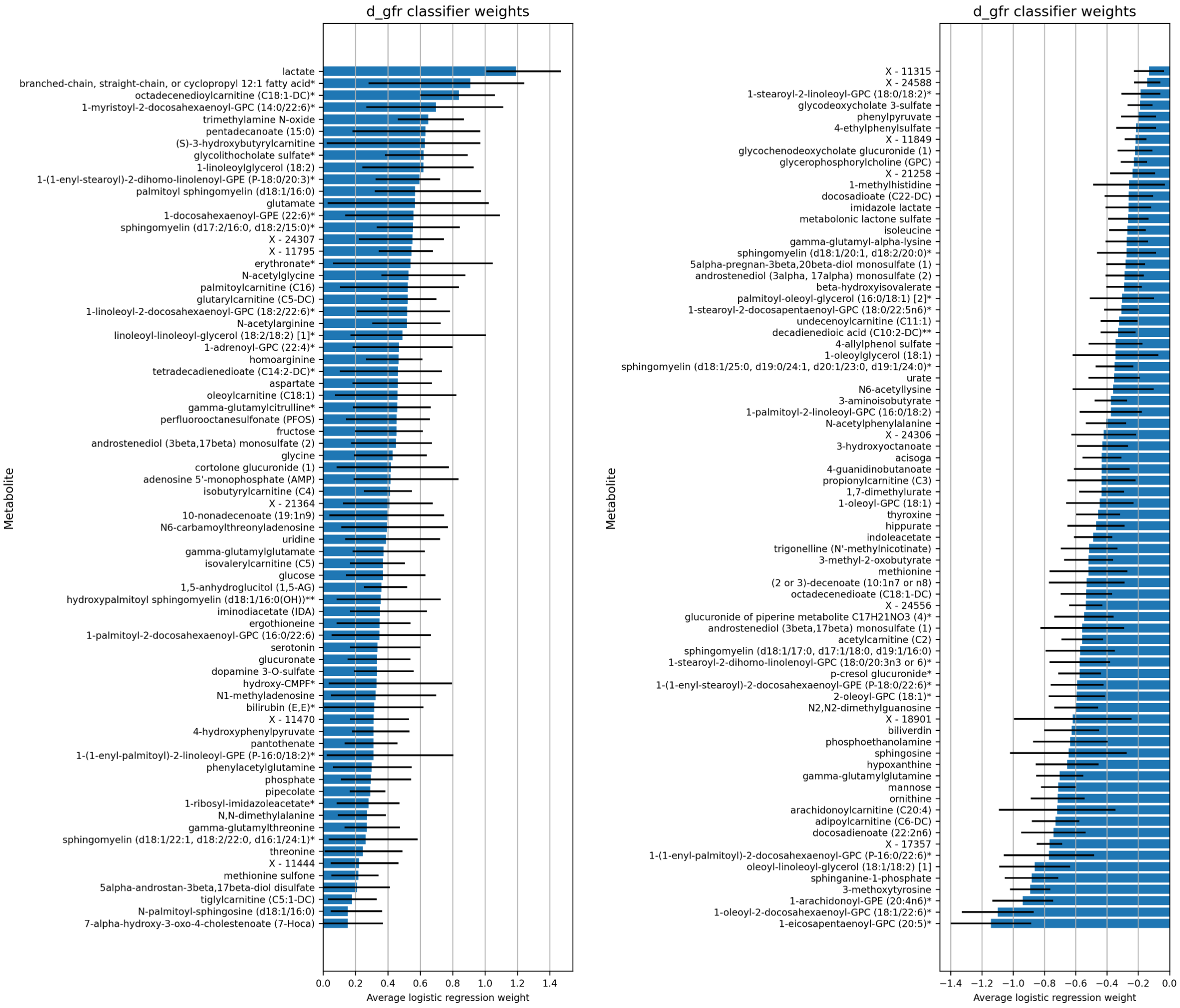

Supplementary Figure 2: All nonzero coefficients for the deGFR logistic regression predictors at 6 months, using the clinical, proteomic, and metabolomic feature sets. The bars indicate the range of coefficient values across all cross-validation runs.

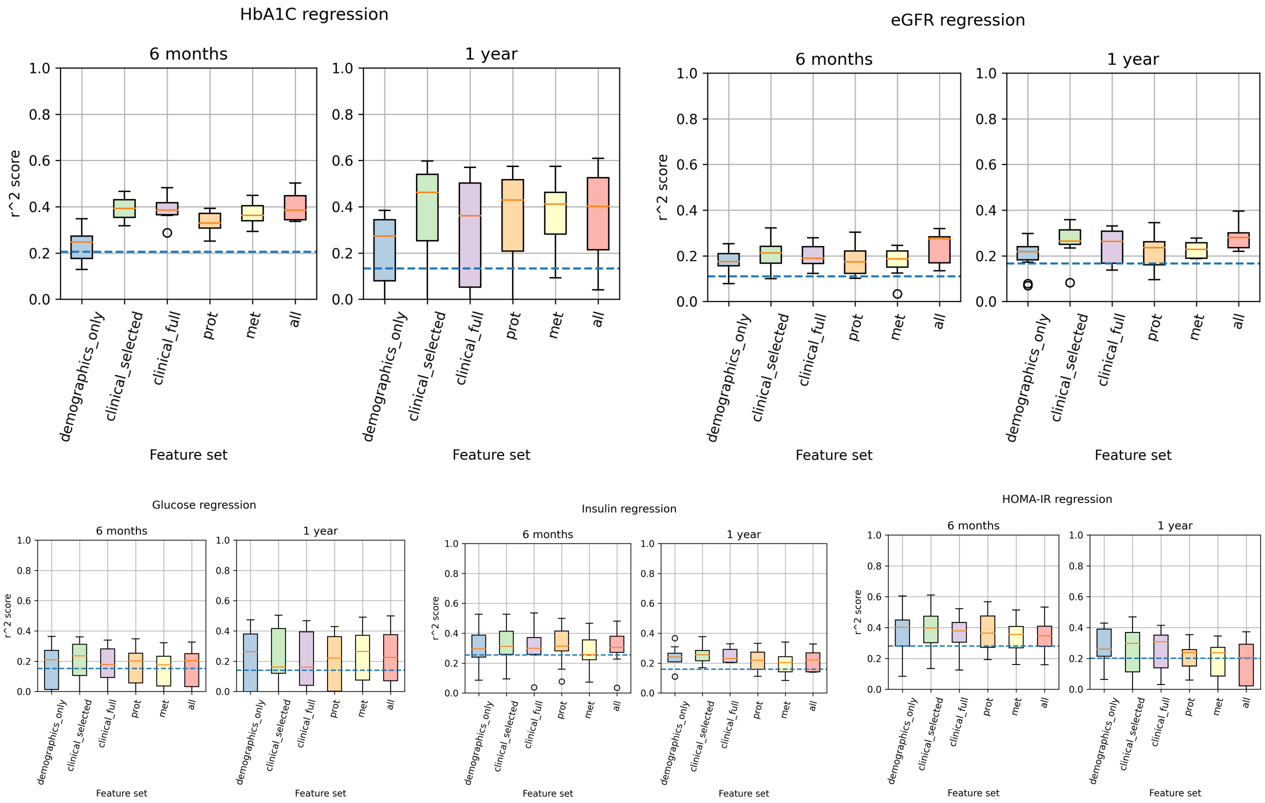

Supplementary Figure 3: Prediction results for changes in HbA1C, eGFR, Glucose, Insulin, and HOMA-IR, using a LASSO regression model for predicting the delta values.

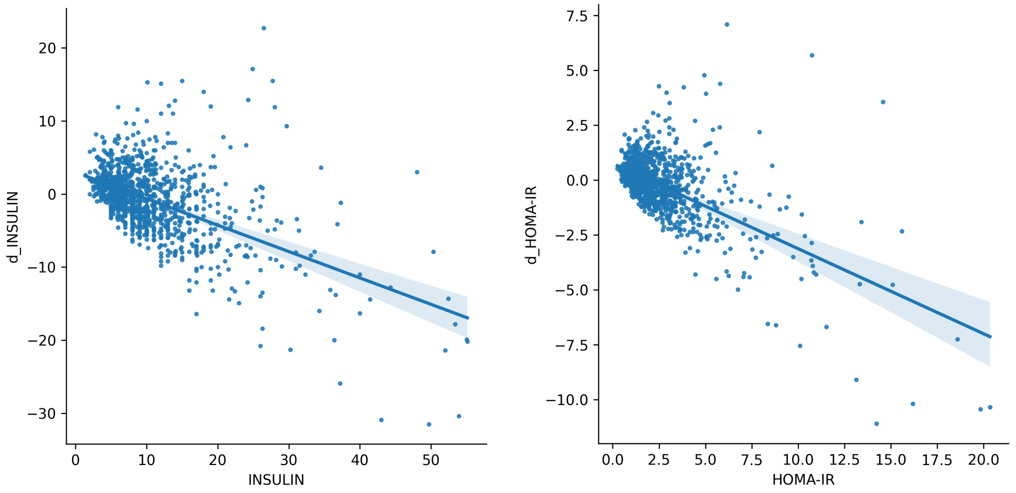

Supplementary Figure 4: Insulin and HOMA-IR changes vs baseline at 6 months. The R^2 values are 0.251 and 0.196, respectively, while the coefficients are -0.1725 and -0.2168.

2.2 Supplementary Tables

Regression models	
**Model name**	**Description**
ElasticNet	ElasticNet linear regression model, fixed regularization parameter
LassoCV	LassoCV model from scikit-learn - LASSO regression with cross-validation to determine the regularization parameter.
ElasticNetCV	ElasticNetCV model from scikit-learn - ElasticNet regression with cross-validation to determine the regularization parameters.
Ridge	Ridge regression model
RidgeCV	RidgeCV model from scikit-learn - Ridge regression with cross-validation to determine the regularization parameters.
SVR	Support vector regression, with RBF kernel
LinearSVR	Linear support vector regression
RandomForestRegressor	Random forest regression
KNeighborsRegressor	k-nearest neighbors regression
**Classification models**	
LogReg	Logistic regression with L2 regularization, regularization parameter fixed at 1 (default parameters)
LogRegLasso	Logistic regression with L1 regularization, regularization parameter fixed at 1
LogRegLassoCV	LogisticRegressionCV - L1-regularized logistic regression with cross-validation to determine the regularization parameter.
LogRegElasticNetCV	LogisticRegressionCV - L1 and L2-regularized logistic regression with cross-validation to determine the regularization parameter.
RidgeClassifier	RidgeClassifier
RidgeClassifierCV	RidgeClassifierCV model from scikit-learn - Ridge classifier with cross-validation to determine the regularization parameters.
SVC	Support vector classifier with an RBF kernel
LinearSVC	Linear support vector classifier
RandomForestClassifier	Random forest classifier
KNeighborsClassifier	k-nearest neighbors classifier

Supplementary table 4: Machine learning models used for predicting changes in clinical variables.
